# A stable and strongly ferromagnetic Fe_17_O_10_^–^ cluster with an accordion-like structure

**DOI:** 10.1038/s42004-023-00952-z

**Published:** 2023-07-13

**Authors:** Lijun Geng, Xiaohu Yu, Zhixun Luo

**Affiliations:** 1 Beijing National Laboratory for Molecular Sciences (BNLMS), State Key Laboratory for Structural Chemistry of Unstable and Stable Species, Institute of Chemistry, Chinese Academy of Sciences, Beijing, 100190 China; 2 Institute of Theoretical and Computational Chemistry, Shaanxi Key Laboratory of Catalysis, School of Chemical & Environment Sciences, Shaanxi University of Technology, Hanzhong, 723000 China; 3 School of Chemistry, University of Chinese Academy of Sciences, Beijing, 100049 P.R. China

**Keywords:** Physical chemistry, Magnetic materials, Magnetic properties and materials

## Abstract

Isolated clusters are ideal systems for tailoring molecule-based magnets and investigating the evolution of magnetic order from microscopic to macroscopic regime. We have prepared pure Fe_*n*_^–^ (*n* = 7-31) clusters and observed their gas-collisional reactions with oxygen in a flow tube reactor. Interestingly, only the larger Fe_*n*_^–^ (*n* ≥ 15) clusters support the observation of O_2_-intake, while the smaller clusters Fe_*n*_^–^ (*n* = 7-14) are nearly nonreactive. What is more interesting is that Fe_17_O_10_^–^ shows up with prominent abundance in the mass spectra indicative of its distinct inertness. In combination with DFT calculations, we unveil the stability of Fe_17_O_10_^–^ within an interesting acordion-like structure and elucidate the spin accommodation in such a strongly ferromagnetic iron cluster oxide.

## Abstract (web summary)

Iron oxide nanoclusters have high potential for high-density energy storage and spintronics microdevices, but systems that are both stable and display strong ferromagnetism remain challenging to obtain. Here, the authors study the gas phase collision reactions of the iron clusters Fe_n_^-^ (n = 7-31) and observe the stable, strongly ferromagnetic iron oxide cluster Fe_17_O_10_^-^ featuring an accordion-like structure.

## Introduction

Magnetic iron oxide nanoparticles are well-known for their significance in targeted drug delivery^1–3^, magneto-responsive therapy and multimodal imaging, as well as microwave-absorption and magneto-optical crossover applications^4–6^. Sub-nano clusters of iron oxides could exhibit altered properties in comparison with larger nanoparticles, enabling highly tuneable magnetism and chemical activity, and thus are ideal candidates for high-density storage or spintronics microdevices when embedded in semiconductors. On the premise and aim of a full understanding of the spin-exchange interactions and structure-property relationship^7,8^, ongoing continuous efforts have also been devoted to understanding the gas-phase reactivity of such clusters^9^, as well as fundamental mechanisms and potential applications relating to metalloenzymes and metallic corrosion and catalysis^10,11^.

Iron compounds could exhibit a variety of magnetic properties, with appropriate nuclearity and topology to function as molecular magnets^12,13^. Typically, FeO is antiferromagnetic at low temperatures but with a high-spin ^5^Δ ground state based on the DFT-GGA calculations^14^; Fe_2_O_3_ is antiferromagnetic but Fe_3_O_4_ is known to be ferrimagnetic. Meanwhile, oxo, peroxo, and superoxo isomers^15^, dioxygen, and oxygen complexes Fe(O_2_)_*n*_ (refs. ^16–18^) and oxygen-rich clusters^19–22^, as well as sequential stoichiometries of (FeO)_*m*_ (ref. ^23^), Fe_*m*_O_*m*+1,2_ (refs. ^23–28^), (Fe_2_O_3_)_*n*_ clusters^29–31^ and (Fe_2_O_3_)_*k*_FeO^+^ (ref. ^32^) clusters have been extensively studied^33–35^. Studies of such unique stoichiometries not only elucidate varying electronic and geometric structures of iron oxides^36^, but also provide fundamental information on the kinetics and thermodynamics of coordination and catalytic reactions^37–43^. Notably, iron atoms in most of these oxide clusters, except a few (FeO)_*n*_ clusters^23^, are separated by oxygen atoms but without direct Fe–Fe bonds, resulting in a tendency of antiferromagnetic rather than ferromagnetic properties.

It has been recognized that the magnetic moments of metal clusters depend on geometry and electron localization^44,45^. Reasonable research interest has been devoted to polynuclear clusters^46–50^, especially the magic-number iron clusters and iron oxide clusters such as a ring cluster Fe_10_ (ref. ^51–53^), cubic Fe_13_O_8_ and cage Fe_12_O_12_ clusters^54–61^. However, the antiferromagnetic states were often found to be much more stable than the ferromagnetic counterparts^61,62^. There seems an incompatible contradiction for iron oxide clusters to bear high stability and strong ferromagnetism.

Recently utilizing the customized deep-ultraviolet laser ionization mass spectrometry technique, the reactions of neutral Co_*n*_ clusters with oxygen were studied. It was found that Co_13_O_8_ dominates the mass distribution in sufficient collisional reactions with O_2_. The distinctive stability of neutral Co_13_O_8_ was demonstrated as a class of metalloxocubes^63^. The prominent mass abundances of Ni_13_O_8_^+^ and Fe_13_O_8_^+^ were also verified in such a flow tube reactor^64,65^. Notably, the cubic M_13_O_8_^+^ (M = Fe, Co, Ni, Rh) clusters prefer high-spin states, and the spin multiplicity increases from Ni (3d^8^4s^2^) to Co (3d^7^4s^2^) and Fe (3d^6^4s^2^), shedding light on the importance of spin accommodation. Likely due to the strong magnetic effect, there were challenges to observe and monitor the reactions of anionic iron clusters.

Until very recently we have made a breakthrough in the preparation of pure Fe_n_^–^ clusters, which enables us to meticulously study their reactions and probe the likely magic-number clusters. Here we observe the gas-phase reactions of the iron clusters Fe_*n*_^–^ (*n* = 7–31) with oxygen and find a stable, strongly ferromagnetic iron oxide cluster Fe_17_O_10_^–^ featuring an accordion-like structure.

## Results and discussion

### Mass spectrometry observation

**Fig. 1:**
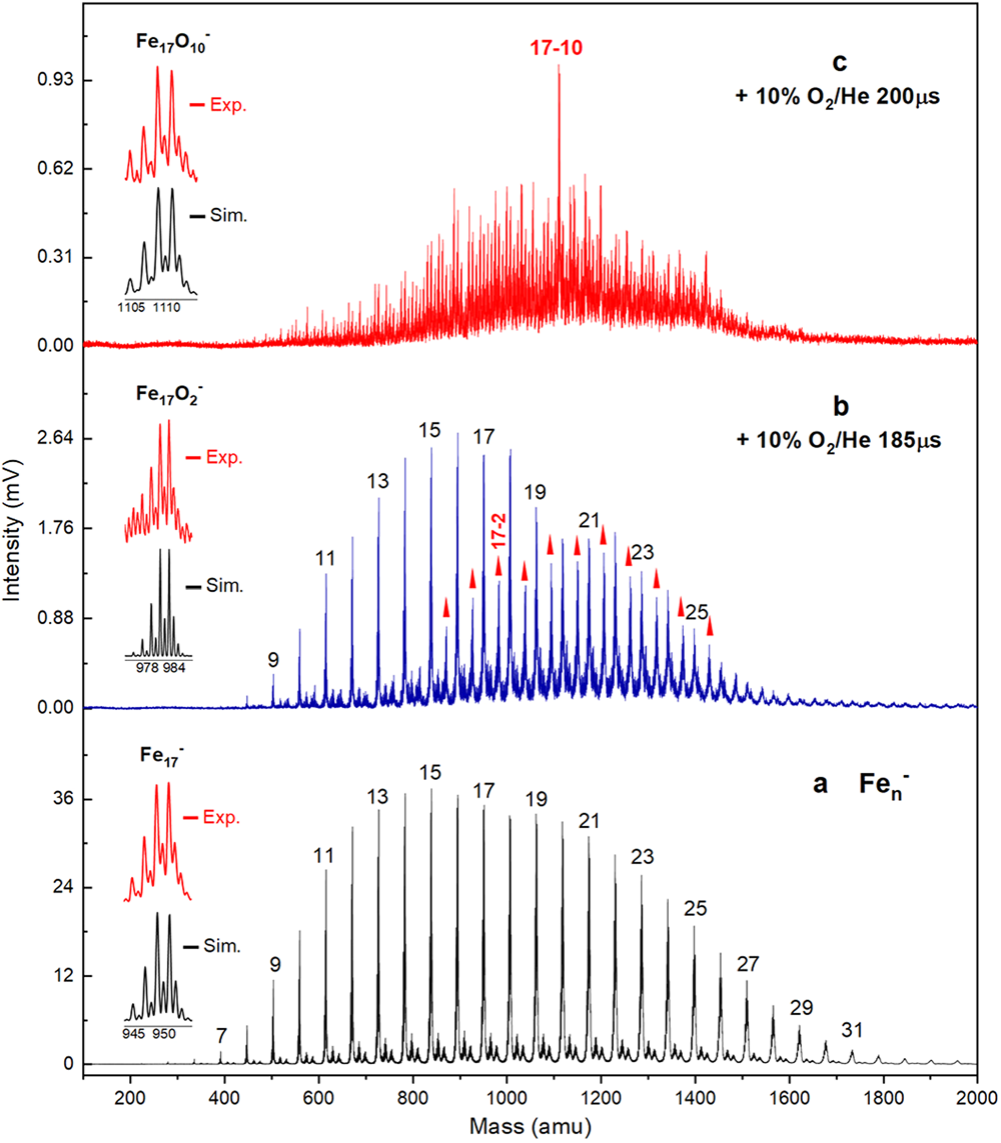
Mass spectrometry observation. **a** TOF mass spectra of the Fe_*n*_^–^ clusters produced by the homemade LaVa source. **b**, **c** Mass spectra of the Fe_*n*_^–^ clusters after exposure to reactions with different amounts of O_2_ (10% in He, 0.1 MPa) corresponding to the on-time of the pulse valve at 185 μs and 200 μs respectively (namely, the reactant molecule density at 2 × 10^19^ and 4 × 10^19^ molecules per cubic meter). The instantaneous pressure of the carrier gas inside the flow tube reactor was ~35 Pa, while the lasting time of reaction gas in the reaction tube is estimated to be 1 ms. The vacuum in the source chamber and TOF chamber at ~4.0  × 10^−3 ^Pa and ~1.0 × 10^−5 ^Pa respectively. The inset shows the simulated and experimental isotope distribution of Fe_17_O_10_^–^, Fe_17_O_2_^–^, and Fe_17_^–^ respectively.

Figure 1 presents the typical mass spectra of the anionic Fe_*n*_^–^ (*n* = 7-31) clusters in the absence and presence of different doses of oxygen reactants (10% in He), corresponding to the on-time of the pulse valve at 185 μs and 200 μs respectively. These bare Fe_*n*_^–^ clusters display a nearly Gaussian distribution of their mass abundances centered at *n* = 16; however, the oxidation products were observed first for the larger Fe_*n*_^–^ (*n* = 15–27) clusters, and the formed Fe_*n*_O_2_^–^ clusters also display a Gaussian distribution centered at *n* = 20 (Fig. 1b). It is supposed that the cross-section of the larger Fe_*n*_^–^ (*n* ≥ 15) clusters benefit the O_2_ adsorption and thermal balance by sufficient collisions with helium; meanwhile, the larger Fe_*n*_^–^·O_2_ clusters could allow for more flexible vibrational structure relaxation to disperse the energy gain of O_2_-adsorption. In comparison, the smaller Fe_*n*_^–^ clusters (*n* ≤ 14) may be subject to a smaller vibrational density-of-states (DOS) and shorter lifetime of the vibrational excitation states thus allowing for fragmentation of the likely formed Fe_*n*_^–^·O_2_ intermediates. With a further increased dose of the O_2_ reactant (details in Supplementary Figs. 1–3), oxygen-rich products emerge in the mass spectra; interestingly, Fe_17_O_10_^–^ shows up with prominent mass abundance relative to all the other Fe_*n*_^–^ and Fe_*n*_O_*m*_^–^ clusters (Fig. 1c). It is anticipated that the reactions of Fe_17_^–^ clusters likely undergo fast stepwise oxidation than the others, along with incidental conversion between unstable and stable species, which can be written as,

1 $${{{{{{\rm{Fe}}}}}}}_{n}^{-}+{{{{{{\rm{x}}}}}}\,{{{{{\rm{O}}}}}}}_{2}\to {{{{{{\rm{Fe}}}}}}}_{n}{{{{{{\rm{O}}}}}}}_{2}^{-}\to {{{{{{\rm{Fe}}}}}}}_{n}{{{{{{\rm{O}}}}}}}_{4}^{-}\to \cdots \to {{{{{{\rm{Fe}}}}}}}_{n}{{{{{{\rm{O}}}}}}}_{10}^{-}$$

2 $${{{{{{\rm{Fe}}}}}}}_{n}{{{{{{\rm{O}}}}}}}_{x}^{-}+{{{{{{\rm{Fe}}}}}}}_{m}+{{{{{\rm{He}}}}}}\to {{{{{{\rm{Fe}}}}}}}_{n+m}{{{{{{\rm{O}}}}}}}_{{{{{{\rm{x}}}}}}}^{-}+{{{{{\rm{He}}}}}}$$

This observation of size dependence is, to some extent, consistent with the previous studies on gas-phase reactivity and interactions of cations and neutral iron clusters. For example, early studies by Whetten et al.^66,67^ showed that the initial stages of ion cluster oxidation favor the formation of cluster dioxides, but a diversity of Fe_*n*_O_*m*_ may be formed as the O_2_ pressure increases. Andersson et al.^68^ found that small Fe_*n*_ clusters are unreactive toward D_2_, but the larger Fe_*n*_ (*n* ≥ 23) do react with a low rate. Griffin and Armentrout^69^ reported a study on the reactions of Fe_*n*_^+^ (*n* = 2–18) with O_2_ by guided ion-beam apparatus and showed that the dioxide cluster cations are dominant products for the larger ones. The size-dependent cross-section for dehydrogenation of ethylene on the cationic Fe_*n*_^+^ (*n* = 2–28) clusters was also studied by Ichihashi et al.^70^, showing that the dehydrogenation reaction increases rapidly above a certain cluster size. It is inferred that the dramatic size effect of iron clusters is associated with the Fe-Fe bonding and variable 3*d* electrons and complex spin-exchange interactions.

### Structure determination

**Fig. 2:**
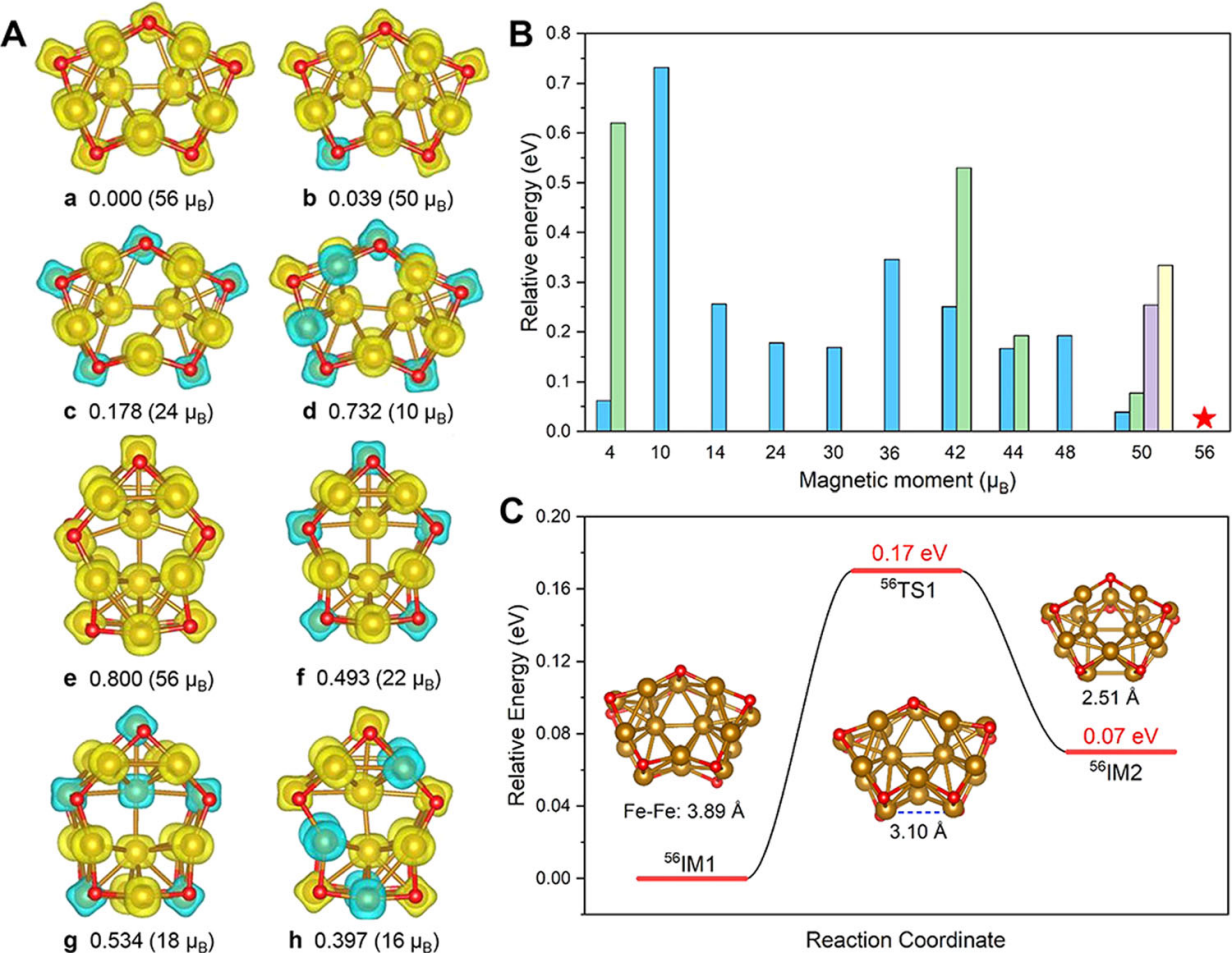
Isomers and magnetic moments. **A** The typical structure isomers and electronic spin-state isomers of Fe_17_O_10_^–^. The ab initio evolutionary algorithm USPEX combined with VASP package was used for the global minima search of the cluster structures. Given the strong electron correlation and likely high-spin states, GGA + U approaches were employed for the calculations to determine the ground state structure of Fe_17_O_10_^–^. **B** The relative energy of Fe_17_O_10_^–^ at the same geometry as the lowest energy structure but varying magnetic moments. **C** Resonating structure isomers of the ground-state ^56^Fe_17_O_10_^–^.

To understand the prominent mass abundance of Fe_17_O_10_^–^, we have performed a global structure search by USPEX employing the VASP software package. The typical structure isomers and electronic spin-state isomers of the Fe_17_O_10_^–^ are shown in Fig. 2A. To our surprise, the lowest energy structure of Fe_17_O_10_^–^ (C_2v_) has 10 μ_3_-O atoms capped at the 10 hollow sites of Fe_17_ which, however, exhibits a different geometry compared with the C_3v_ structure of nascent Fe_17_^–^ (Fig. 2A-a). The sub-nano Fe_17_O_10_^–^ cluster can be viewed as the fusion of two Fe_13_O_8_ cubic units (with an overlapped Fe_9_O_6_, Supplementary Fig. 9). Notably, both M_13_O_8_ and M_9_O_6_ (M = Fe, Co, Ni) correspond to magic numbers in the transition-metal oxide clusters^48^.

What is more interesting is that the ground state of Fe_17_O_10_^–^ finds an extremely high magnetic moment (56 μ_B_, i.e., 55 spin-unpaired electrons totally). Considering that the Fe atom has multiple spin-unpaired electrons and the ground state of O_2_ is in a triplet state, we have compared the energy of Fe_17_O_10_^–^ at the GGA + U method with a variety of magnetic moments being considered (from 4 to 56 μ_B_), as shown in Fig. 2B. The results verified that the ground state of Fe_17_O_10_^–^ corresponds to the accordion-like C_2v_ structure with a magnetic moment of 56 μ_B_. Notably, the spin magnetic moment of Fe_17_^–^ cluster is estimated to be 54 μ_B_ (i.e., 53 spin-unpaired electrons totally), indicative of spin-excitation to form the strongly ferromagnetic Fe_17_O_10_^–^ (55 spin-unpaired electrons totally). Within this high-spin state, the accordion-like C_2v_ structure allows for a resonating structure isomer of 0.07 eV higher in energy (Supplementary Figs. 7-8). The vibrational structure relaxation within two resonating isomers undergoes a small energy barrier of 0.17 eV (Fig. 2C), which is reminiscent of playing the accordion.

### Relative stability

**Fig. 3:**
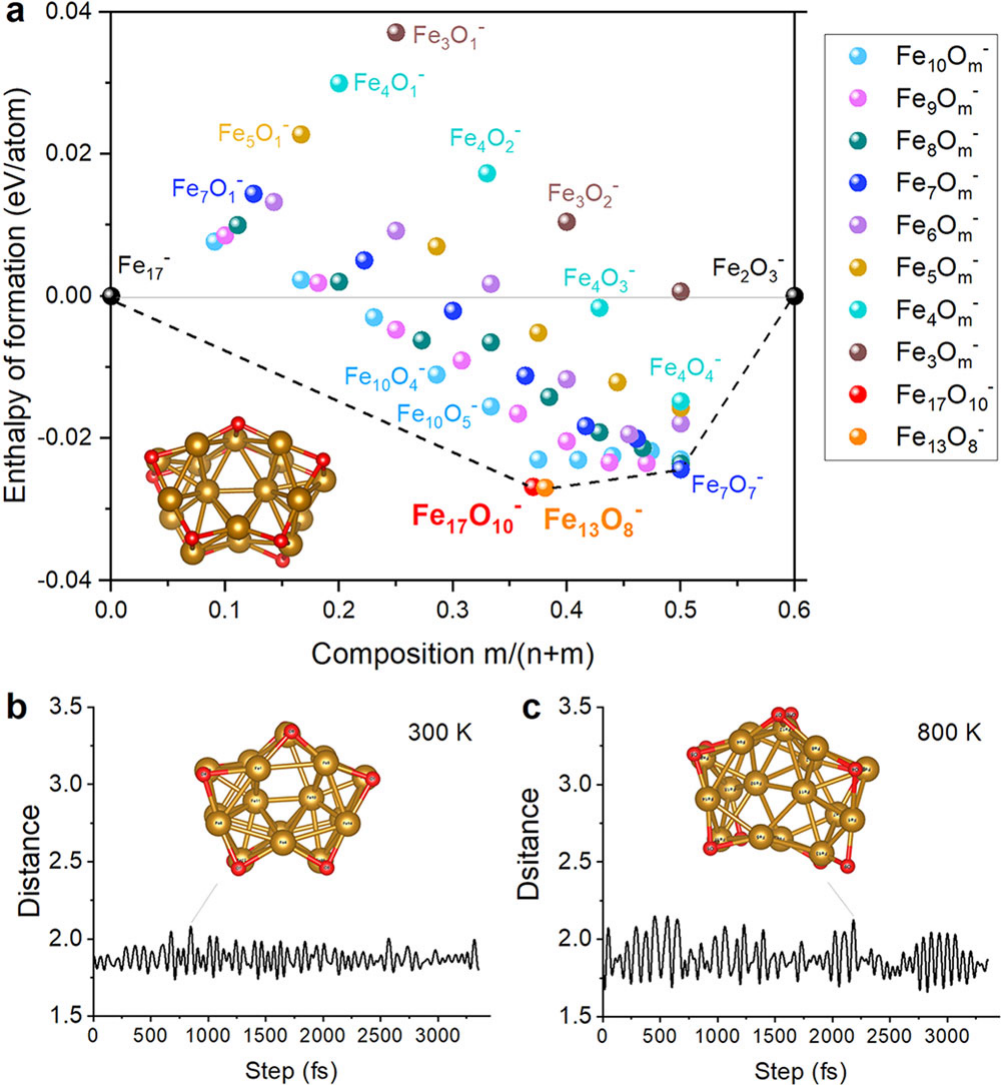
Thermodynamic phase diagram and AIMD analysis. **a** Relative ground state formation enthalpies per atom of all the studied Fe_*n*_O_*m*_^–^ clusters within a convex hull (with respect to Fe_17_^–^ and Fe_2_O_3_^–^), the points of the set (x, y) are given by ${{{{{\rm{x}}}}}}=\frac{m}{n+m}{{{;}}}{\ y}=[{E}_{F{e}_n{{O}_{m}}^{-}}-m\cdot \frac{{E}_{F{e}_{2}{{O}_{3}}^{-}}}{3}-\left(n-\frac{2}{3}m\right)\cdot \frac{{E}_{F{{e}_{17}}^{-}}}{17}]/(n+m)$, corresponding to the total energy of Fe_*n*_O_*m*_^–^ clusters. The *x*-axis refers to the atomic number ratio of O relative to the total, while the *y*-axis shows the relative formation enthalpies per atom above the hull. The cluster structures are given in Supplementary Fig. 6. **b**, **c** The AIMD simulations of Fe_17_O_10_^–^ at 300 and 800 K for 3250 fs, with the Fe1-O4 distance indicated in Å. The time step was set to 1 fs.

We have compared the relative stability of a variety of Fe_*n*_O_*m*_^–^ clusters. Figure 3a plots a thermodynamic phase diagram by involving all the studied Fe_*n*_O_*m*_^–^ clusters within a convex hull (structural details in Supplementary Figs. 4–6) by borrowing ideas of such phase-diagram used in describing the thermodynamic stability of metal alloys and doped compounds^71,72^. For these Fe_*n*_O_*m*_^–^ clusters, the *x*-axis is set to the ratio of O and Fe atoms within a certain Fe_*n*_O_*m*_^–^ cluster, and the y-axis corresponds to the relative formation enthalpies per atom above the hull. On this basis, in the left-side axis of this convex hull, we have the 1/*n* energies of the Fe_*n*_^–^ clusters; while in the right-side axis, we plot the 1/5 *m* energies of (Fe_2_O_3_)_*m*_ clusters. When connecting the convex polygon with all the Fe_*n*_O_*m*_^–^ clusters being involved, Fe_17_O_10_^–^ and Fe_13_O_8_^–^ are both located in the lowest position (Fig. 3a), indicative of their prominent thermodynamic stability. In addition, we also conducted ab-initio molecular dynamics (AIMD) simulations and find that the structure of Fe_17_O_10_^–^ is undissociated up to 800 K (Figs. 3b and 3c, also Supplementary Fig. 10), verifying its outstanding thermal stability.

### Reaction mechanism

**Fig. 4:**
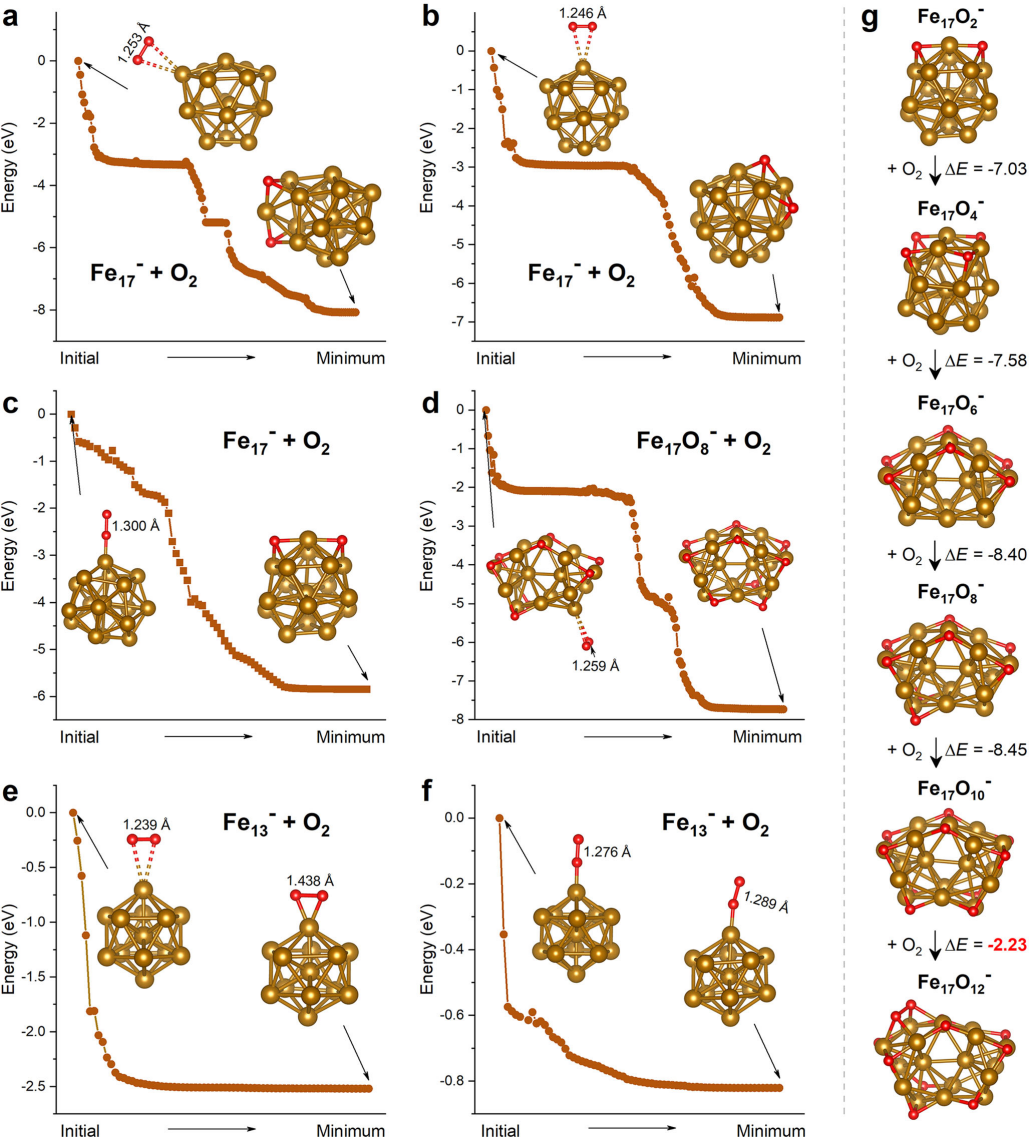
Reaction pathway. **a**–**c** The typical reaction model of oxygen on the ground-state Fe_17_^–^cluster. **d**, The typical formation of Fe_17_O_10_^–^ through Fe_17_O_8_^–^ reacting with O_2_. **e**, **f** The typical reaction model of oxygen on the ground-state Fe_13_^–^ cluster with the end-on and side-on orientation. **g** Thermodynamic energy changes from Fe_17_O_2_^–^ to Fe_17_O_12_^–^ [ΔE = E(Fe_17_O_2x+2_^–^)– E(Fe_17_O_2x_^–^)– E(O_2_)]. All the optimization was conducted by applying the Gaussian 09 software package, with the BPW91 and the 6-311 G(d) basis set for both Fe and O atoms.

Considering the prominent mass abundance of Fe_17_O_10_^–^ (instead of cubic Fe_13_O_8_^–^) observed in the mass spectrometry experiments, we further analyzed the reaction processes of Fe_17_^–^ and Fe_13_^–^ with oxygen by DFT calculations based on the Gaussian software package. As shown in Fig. 4a–c, when an oxygen molecule attacks Fe_17_^–^ in the side-on or end-on orientation, the O_2_ molecule dissociates spontaneously giving rise to two μ_3_-O atoms capped on the neighboring hollow sites of the Fe_17_^–^ cluster. We also tested Fe_17_O_8_^–^ + O_2_ (Fig. 4d) and found the similar spontaneous O–O dissociation toward the formation of Fe_17_O_10_^–^. In contrast, oxygen molecules adsorbed on the surface of Fe_13_^–^ in a side-on or end-on manner do not undergo spontaneous dissociation (Fig. 4e, f), although the end-on adsorption (superoxo-state) may transform to peroxide state ^40^IM1 (i.e., undissociated O_2_ bonding to two Fe atoms) giving rise to likely O–O dissociation at a small energy barrier (Supplementary Fig. 15). Also, we have calculated the thermodynamic energy changes for the O_2_-addition to the Fe_17_O_n_^–^ (*n* = 2, 4, 6, 8) clusters, as shown in Fig. 4g. Interestingly, the energy gain for Fe_17_O_10_^–^ to Fe_17_O_12_^–^ (−2.23 eV) is much smaller than that for each step of Fe_17_O_2_^–^ → Fe_17_O_4_^–^ → Fe_17_O_6_^–^ → Fe_17_O_8_^–^ → Fe_17_O_10_^–^, indicating that the formation of Fe_17_O_10_^–^ is significantly faster than its subsequent conversion to Fe_17_O_12_^–^. This could be the reason why Fe_17_O_10_^–^ instead of Fe_13_O_8_^–^ is dominated in the reactions of Fe_*n*_^–^ clusters with oxygen. In addition, the DFT calculation results show that Fe_11_^–^ also allows for oxygen addition and O–O dissociation (Supplementary Figs. 13–14) but no such products were observed in our mass spectrometry experiments. It is supposed that the chemical activity or inertness is not just determined by the binding energy but may include a comprehensive factor of the stability/activity of both the nascent cluster and product, as well as the dynamics of reaction and conversion.

### Electronic structure and property

**Fig. 5:**
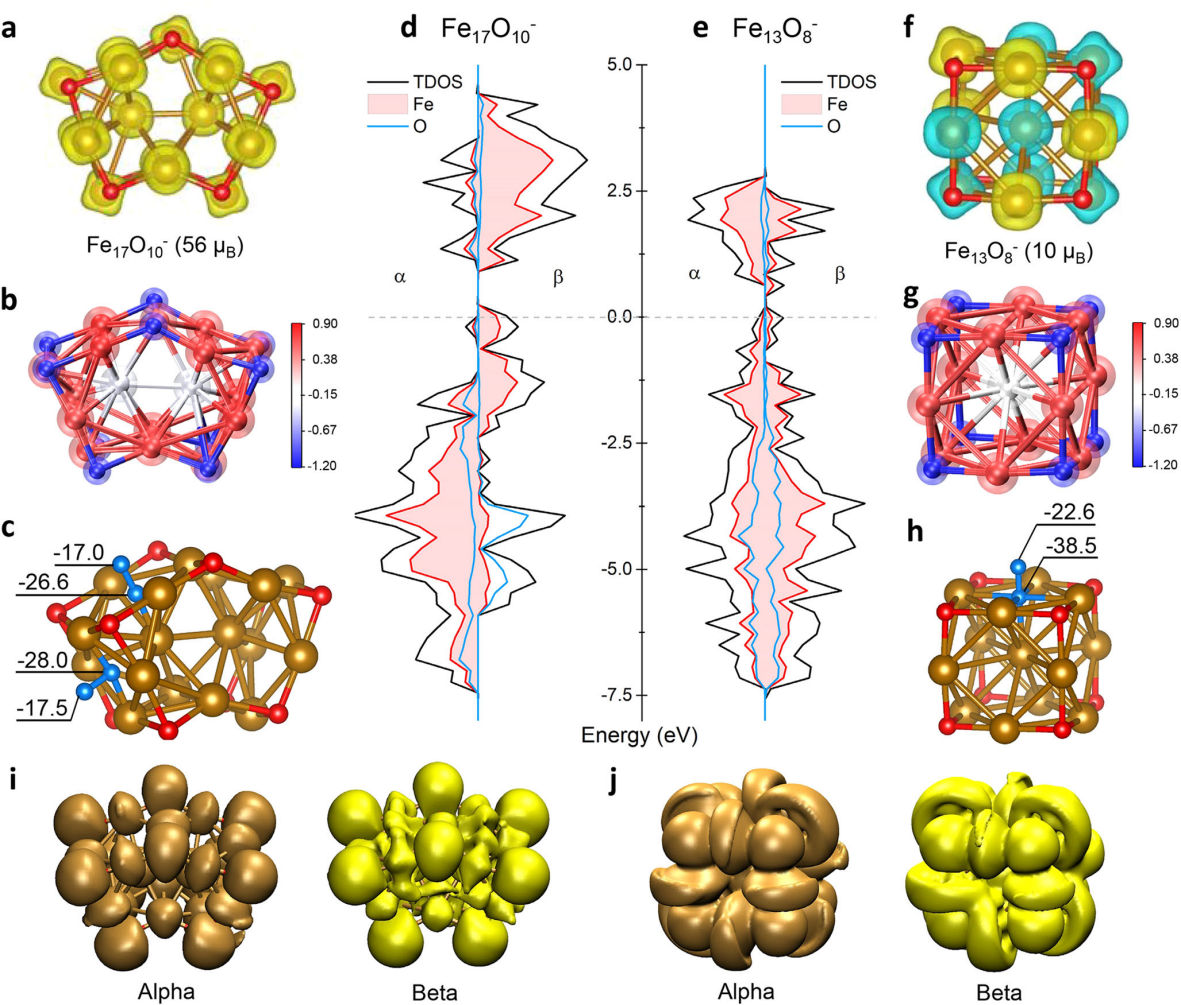
Spin density, charge distribution, ELF and DOS analyses of the Fe_17_O_10_^–^ and Fe_13_O_8_^–^ clusters respectively. **a**, **f** The electronic spin density; **b**, **g** the NPA charge distribution; **c/h**, the calculated nucleus-independent chemical shifts (NICS), with NICS(0) and NICS(1) corresponding to the center and 1.0 Å above the square surface, respectively, performed at the BPW91/6-311 g(d) level of theory. **d**, **e** total and partial density of states (DOS); **i**, **j** 3D-ELF analysis of the alpha and beta electrons in the Fe_17_O_10_^–^ (iso value = 0.25) and Fe_13_O_8_^–^ (iso value = 0.1) respectively.

Notably, Fe_17_O_10_^–^ has a high-spin ground state (56 μ_B_) pertaining to strong ferromagnetism; in comparison, the cubic Fe_13_O_8_^–^ optimized by the same GGA + U approach finds a ground state of ferrimagnetic characteristics (10 μ_B_). To fully understand the novelty behind the two clusters, Fig. 5 presents a comparison of the surface charge distribution by natural population analysis (NPA, details in Supplementary Table 3), the total and partial density of states (DOS), and the nucleus-independent chemical shift (NICS)^73^. It is shown that the NPA charge distributions display similar negative values on all the μ_3_-O atoms (Fig. 5b vs. 5g), embodying the same oxygen bonding mode for the two clusters (detailed bond lengths in Supplementary Table 2).

Also, the NICS values on the quasi-square surfaces of the two clusters are comparable with each other (Fig. 5c vs. 5h); however, the DOSs of Fe_17_O_10_^–^ and Fe_13_O_8_^–^ exhibit remarkable differences (Fig. 5d vs. 5e). For Fe_13_O_8_^–^, the alpha and beta electrons display symmetrical DOS patterns, which is also embodied in the spin density (Fig. 5f); however, there is strong spin polarization in the Fe_17_O_10_^–^ cluster (Fig. 5d), which accounts for the high-spin state of Fe_17_O_10_^–^ with a significantly enlarged α-HOMO-LUMO gap. Interestingly, the majority spin bands (alpha electrons) for the DOSs of Fe_17_O_10_^–^ dominate in the energy range of −2 ~ −7.5 eV, while the dominant minority-spin bands (beta electrons) in the range of 0 ~ 5.0 eV. The strong spin polarization in Fe_17_O_10_^–^ in contrast to Fe_13_O_8_^–^ is also depicted by electron localization function (ELF)^74^ analysis, as shown in Fig. 5i, j (details in Supplementary Figs. 11–12), where the bonding and nonbonding areas of local electron-pair density are displayed, shedding light on the β-aromaticity and mutual compatibility of both strong ferromagnetic property and high stability.

## Conclusions

In summary, utilizing a customized mass spectrometer we studied the reactions of iron clusters with oxygen and observed the prominent inertness of Fe_17_O_10_^–^. Global-minima structure search by USPEX interfaced with the VASP software package has determined the ground state of Fe_17_O_10_^–^ corresponding to a high-spin ferromagnetic accordion-like structure. DFT calculations of thermodynamics and dynamics rationalized its reasonable stability and the feasible reaction dynamics for Fe_17_^–^ to react and form dissociative oxygen addition successfully. The observation of an absence of Fe_13_O_8_^–^ is consistent with the fact that small Fe_*n*_^–^ (*n* = 7–14) clusters are less reactive with oxygen to form stable oxides. We highlight the strong ferromagnetism of Fe_17_O_10_^–^ for sub-nano materials with well-defined components and geometric/electronic structure. Such a strongly ferromagnetic stable cluster could be an ideal candidate for high-density storage or spintronics microdevices.

## Methods

### Experimental methods

The experimental setup is a home-built reflection time-of-flight mass spectrometer (Re-TOFMS), it consists of a customized vacuum system, a mini flow tube reactor ($\varPhi$ = 6 mm, *L* = 60 mm), and a home-built laser vaporization (LaVa) source. In this experiment, the Fe_*n*_^–^ clusters were prepared by ablating a clean and rotating iron disk (99.95%) with a pulsed 532 nm laser (Nd:YAG) at a repetition rate of 10 Hz in the LaVa source. In a nozzle ($\varPhi$ = 1.35 mm, *L* = 35 mm), the clusters were cooled down during a supersonic expansion process by the pulsed He carrier gas (99.999%, 10.0 atm) which was controlled by a pulsed valve (Series 9, General Valve). For reactions between the Fe_*n*_^–^ clusters and O_2_, the reactant gas of 10% O_2_ in He (1.0 atm) was injected into the flow tube reactor (at room temperature) and the pressure of the carrier gas inside this tube was kept at ~35 Pa. More detailed descriptions can be found elsewhere (Supplementary S1. Methods).

### Theoretical methods

The ab initio evolutionary algorithm USPEX^75,76^ combined with VASP package^77–79^, which has been successfully applied to clusters, was used to search the stable Fe_*n*_O_*m*_ clusters. More detailed description of these calculations can be found in the Supporting Information (Supplementary Table 1). In the thermodynamic phase diagram, the geometric structures of Fe_*n*_O_*m*_^–^ (*n* = 3-10, *m* ≤ *n*), Fe_2_O_3_^–^, Fe_13_O_8_^–^, and Fe_17_O_10_^–^ clusters and all possible spin states are optimized by applying the Gaussian 09 software package, with the Becke’s exchange and Perdew−Wang’s correlation functionals (BPW91) and the 6-311 G(d) basis set for all elements (Fe, O). Vibrational frequency calculations and zero-point vibrational corrections were performed for all of the optimization and energy calculations. The transition states (TS) were checked to ensure that there is only one imaginary frequency, and the intrinsic reaction coordinate (IRC) scan was employed to ensure a connection with both intermediates in the reaction pathways. Gibbs free energy were calculated at a temperature of 298 K. The structures and NPA population are plotted by the software package of VESTA and visual molecular dynamics (VMD). More detailed descriptions of the computational methods can be found elsewhere (Supplementary S1. Methods).

## Supplementary information

Peer Review File

Supporting Information

## Data Availability

The data that support the findings of this study are available within the article and its Supplementary Information or from the corresponding author upon reasonable request.
