# Supplementary material for: A stable and strongly ferromagnetic Fe17O10– cluster with an accordion-like structure
Source: Commun Chem. 2023 Jul 13;6:149. doi: 10.1038/s42004-023-00952-z (PMC10345149; doi:10.1038/s42004-023-00952-z)
Supplement: Supplementary file 2 — Supporting Information [file 42004_2023_952_MOESM2_ESM.pdf]

Supplementary Information for

A stable and strongly ferromagnetic $Fe_{17}O_{10}^{-}$ cluster with an accordion-like structure

Lijun Geng[1], Xiaohu Yu[2]* and Zhixun Luo[1,3]*

[1] Beijing National Laboratory for Molecular Sciences (BNLMS), State Key Laboratory for Structural Chemistry of Unstable and Stable Species, Institute of Chemistry, Chinese Academy of Sciences, Beijing 100190, P.R. China;

[2] Institute of Theoretical and Computational Chemistry, Shaanxi Key Laboratory of Catalysis, School of Chemical & Environment Sciences, Shaanxi University of Technology, Hanzhong 723000, P.R. China.

[3] School of Chemistry, University of Chinese Academy of Sciences, Beijing 100049, P.R. China.

*Corresponding authors. Emails: yuxiaohu950203@126.com (XY); zxluo@iccas.ac.cn (ZL).

Table of contents

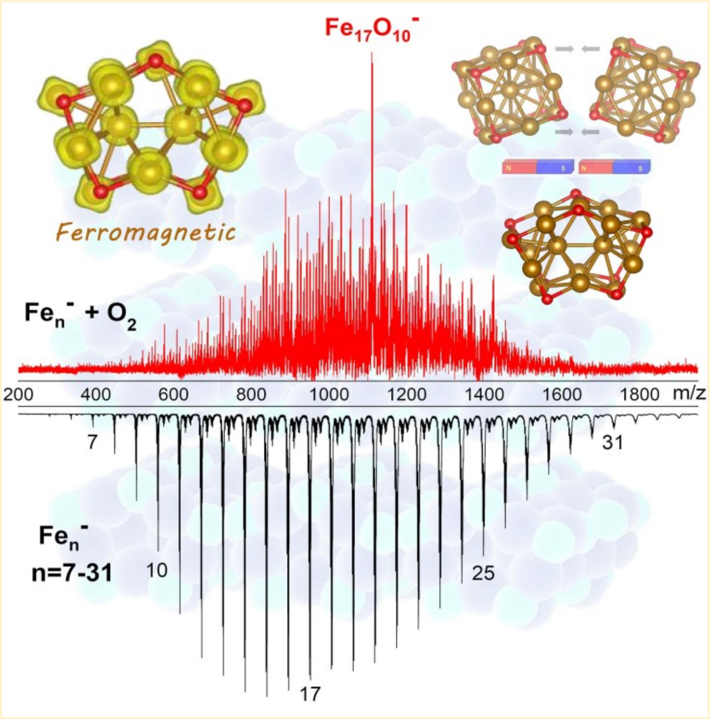

Supplementary S1. Methods 3
Experiemental 3
Computational 3
Supplementary S2. Experimental details 4
Supplementary S3. Computational details 7
Structural determination 7
The other $Fe_nO_m^-$ clusters 8
The electron-spin isomers 9
Geometry and AIMD simulations 10
ELF and NICS analyses 11
Bond lengths and NPA charge 12
O_2-binding analysis 14
References 15

Supplementary S1. Methods

Experiemental

The experimental results leading to the finding of this study was conducted by a customized reflection time-of-flight mass spectrometer (Re-TOFMS). In addition to the homemade vacuum system, we used a compact flow tube reactor (Φ = 6 mm, L = 60 mm) and a 10 Hz pulsed 532 nm laser (Nd:YAG) for the laser evaporation (LaVa). The lasting time of reaction gas in the flow tube is estimated to be 1 ms and the flowing velocity of the carrier He gas is about 1100 m/s. In the typical experiments, the Fe_n^- clusters were prepared by ablating a clean and rotating iron disk (99.95%) with the pulsed 532 nm laser at (~20 mJ pulsed energy), and homemade tubular nozzle (Φ = 1.35 mm, L = 35 mm). The clusters are supposed to be sufficiently cooled down during a supersonic expansion process by the 10.0 atm He carrier gas (99.999%) controlled by a pulsed valve (Series 9, General Valve). For reactions between the Fe_n^- clusters and O_2, the reactant gas of 10% O_2 in He (1.0 atm) was injected into the flow tube reactor and the pressure of the carrier gas inside this tube was about ~35 Pa at T = 298 K. The reactant molecule density in the fast-flow reactor was estimated to be 10^{19}–10^{20} molecules·m^{-3} when the on-time of the pulsed valve was set to 100–200 μs.

Computational

The structure search of stable Fe_nO_m clusters was carried out by using the ab initio evolutionary algorithm USPEX combined with spin-polarized density functional theory (DFT). The first-generation structures of Fe_nO_m clusters were randomly produced with point group symmetry. Since the Fe_nO_m clusters are assumed to have ferromagnetic (FM), antiferromagnetic (AFM), ferrimagnetic (FiM) or nonmagnetic (NM) moments, the corresponding values of magnetic moments of Fe atom in Fe_nO_m clusters are set as 1 for low spin (LS), 4 for high spin state (HS) with the sign (+ or -) depending on FM or AFM, or 0 for NM. For the evolutionary search, either the relaxed energy or the total magnetic moment was used as a criterion (fitness function) for parent structure selection to generate new structures by various evolutionary operators, such as heredity and mutations (lattice mutation, soft-mode mutation, spin mutation). The optimized individual magnetic moments were saved for further data processing by new operators of spin heredity or spin mutations, allowing to find optimal magnetic order. When the first-generation structures are fully relaxed, all the obtained structures of Fe_nO_m are ranked by energy. Then, the new generation of structures was produced from the lower-energy structures in the ranking of the previous one by heredity (40%), softmutation (20%), permutation (10%), spin mutation (10%), the remaining 20% being produced by the random symmetric algorithm. Calculations proceeded for a certain number of generations until the lowest energy structure of Fe_nO_m cluster was found. For all the calculations, the Fe_nO_m clusters were surrounded by a 12-Å-thick vacuum.

The structure relaxation and total energy calculation of the Fe_nO_m clusters were performed by using the projector-augmented wave (PAW) method[1] as implemented in the VASP package[2-4]. The exchange-correlation energy was computed within the generalized gradient approximation (GGA) with the functional of Perdew, Burke, and Erzerhof (PBE)[5], and the 3p, 4s, and 3d electrons of iron and the 2s and 2p electrons of oxygen were treated as valence electrons. To accurately describe the electronic and magnetic properties of iron oxide clusters, an effective $U_{eff} = U{-}J$ value of 4 eV was used for Fe atoms[6, 7] in the DFT+U method[8], which has been shown to provide a good compromise for the description of both atomic and electronic structures of iron oxides. The kinetic energy cutoff of the plane-wave basis was set at 400 eV, and structure relaxations were conducted until the Hellmann–Feynman force on each atom was less than 0.001 eV/Å. Vibrational frequency calculations were conducted to ensure that the predicted structures have no imaginary frequencies which would indicate instability. To understand the stability of $Fe_{17}O_{10}^-$ cluster at different temperatures, we also directly optimized the $Fe_{17}O_{10}^-$ cluster starting from

predicted most stable structure by evolutionary program using molecular dynamics (MD) simulations. The temperatures were kept at 600, 700, 800, and 900 K for 3.5 ps respectively, and the time step was set to 1 fs. Different structures from the MD simulations were optimized and compared with the most stable structure to verify the lowest-energy ground state.

Supplementary S2. Experimental details

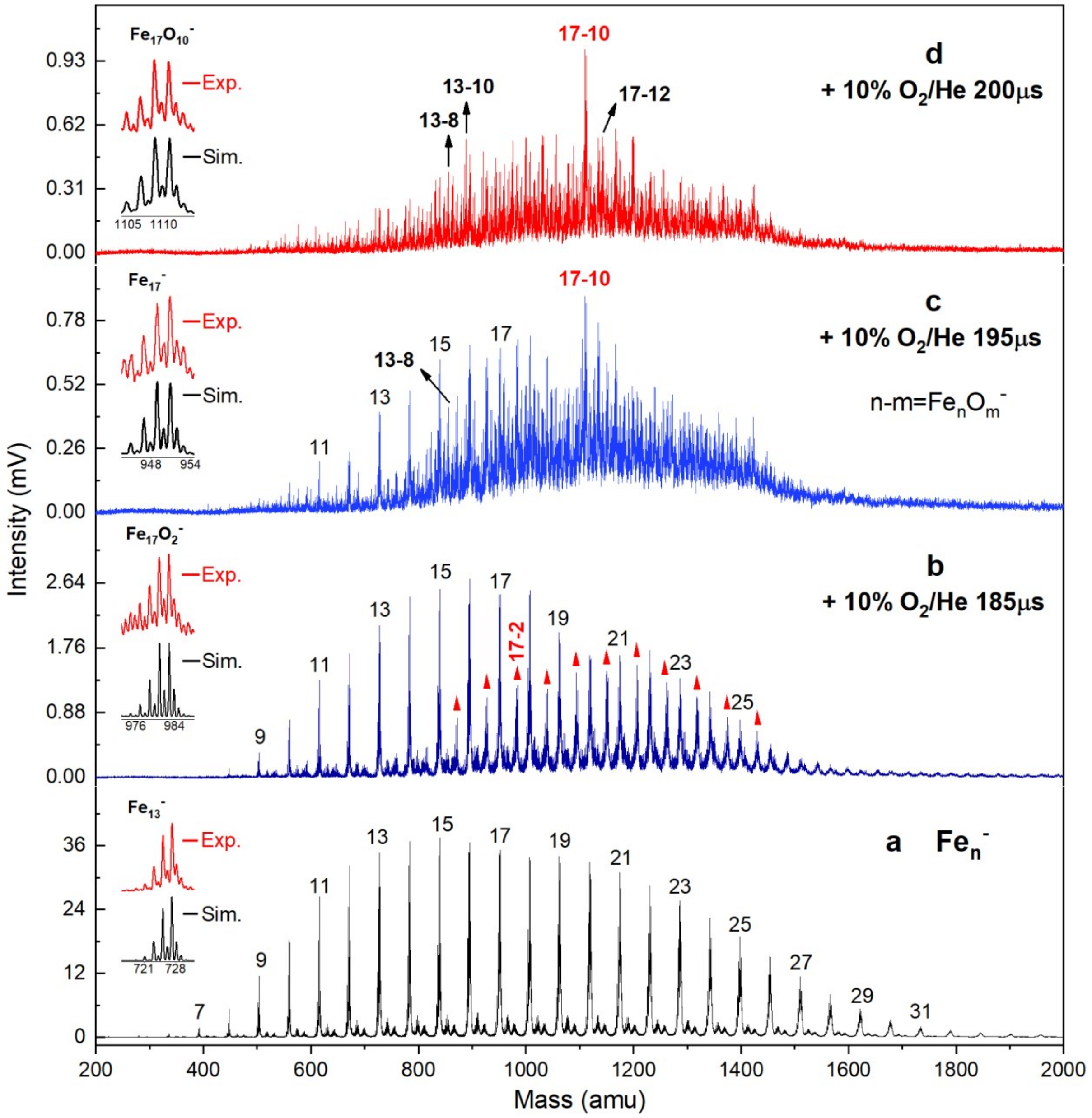

Supplementary Figure 1 | TOF mass spectra of the Fe_n^- clusters produced by the LaVa source **(a)** and after exposure to the reaction with different amount of O_2 (10% in He), with the on-time of the pulse valve at 185 μs **(b)**, 195 μs **(c)**, and 200 μs **(d)**, respectively.

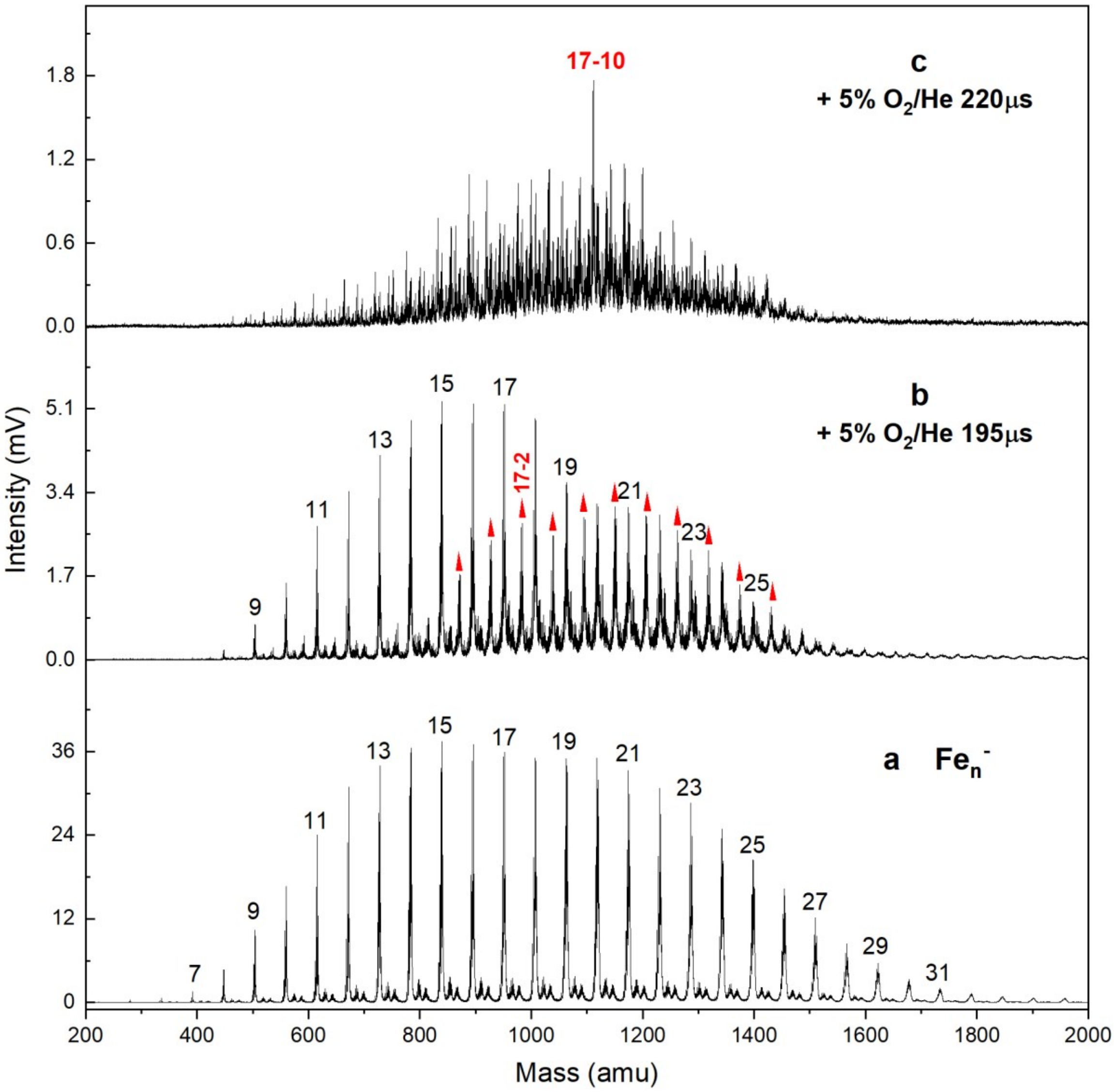

Supplementary Figure 2 | TOF mass spectra of the Fe_n^- clusters produced by the LaVa source **(a)** and after exposure to the reaction with different amount of O_2 (5% in He), with the on-time of the pulse valve at 195 μs **(b)**, and 220 μs **(c)**, respectively.

We have estimated the reaction rate contansts for the Fe_n^- (n = 12−25) clusters with O_2. The observed reaction of "$Fe_n^- + m\,O_2 \rightarrow Fe_nO_2^-$" can be described as a pseudo-first-order reaction, $\ln\left(\frac{I}{I_0}\right) = -\rho k t$, where k corresponds to the reaction rate constant, t refers to the average reaction time, ρ corresponds to the molecular number density of the reaction gas. The calculation details and an experiment-based calibration of the reaction gas molecule density can be found elsewhere.[9]

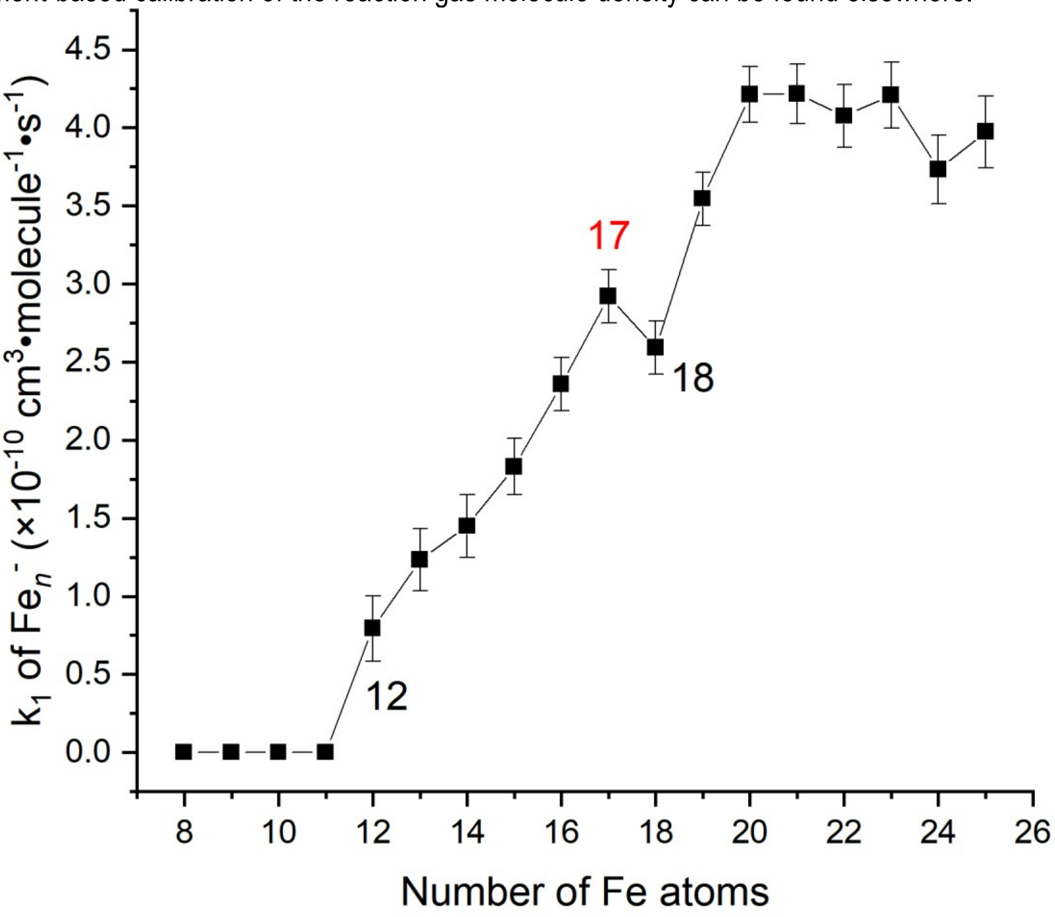

Supplementary Figure 3 | Experimentally determined rate constants k_1 for the reactions between Fe_n^- (n = 12−25) and O_2. The error bars show the uncertainty, corresponding to systematic errors in estimating the integral area of the mass peaks. Considering that Fe_n^- (n = 8−11) are almost not reactive in the mass spectrometry observation, we simply record their k values as zero for a comparison in this study.

Supplementary S3. Computational details

Structural determination

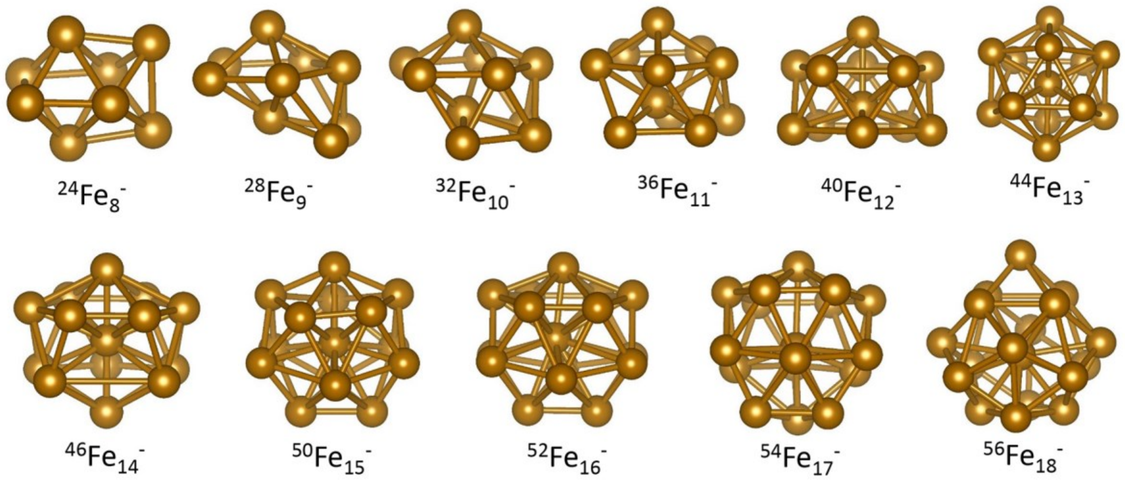

Supplementary Figure 4 | The DFT-optimzied ground-state structures of the Fe_n^- (n = 8−18) clusters at BPW91/6-311g(d) level of theory. The structures are optimized on a basis of the geometries determined by previously reported studies[10]. The presuperscripts represent spin multiplicity.

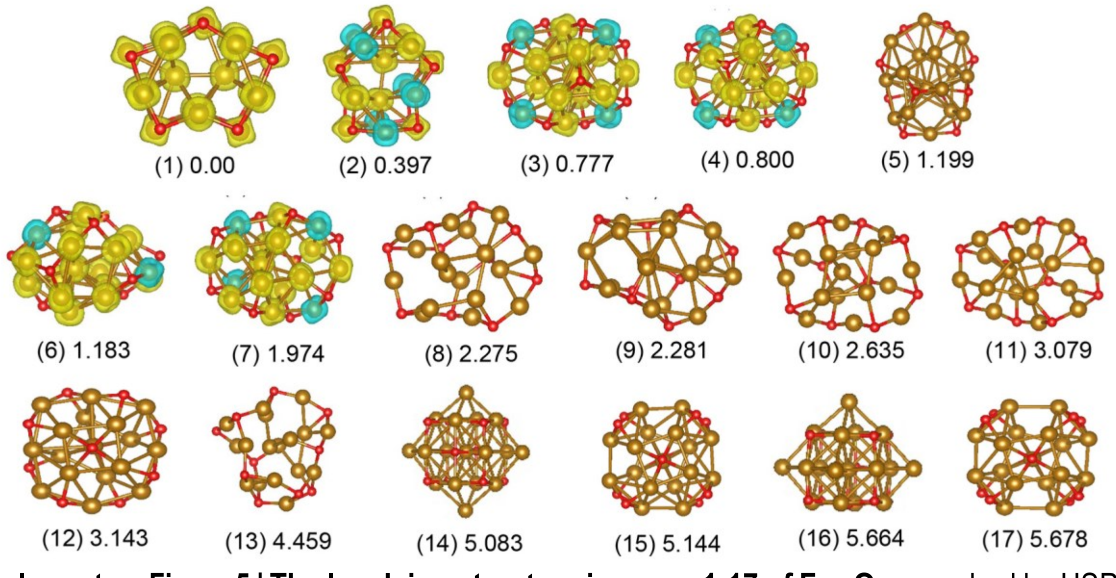

Supplementary Figure 5 | The low-lying structure isomers 1-17 of $Fe_{17}O_{10}$ searched by USPEX invoking the VASP program. The relative energies are given in eV.

The other $Fe_nO_m^-$ clusters

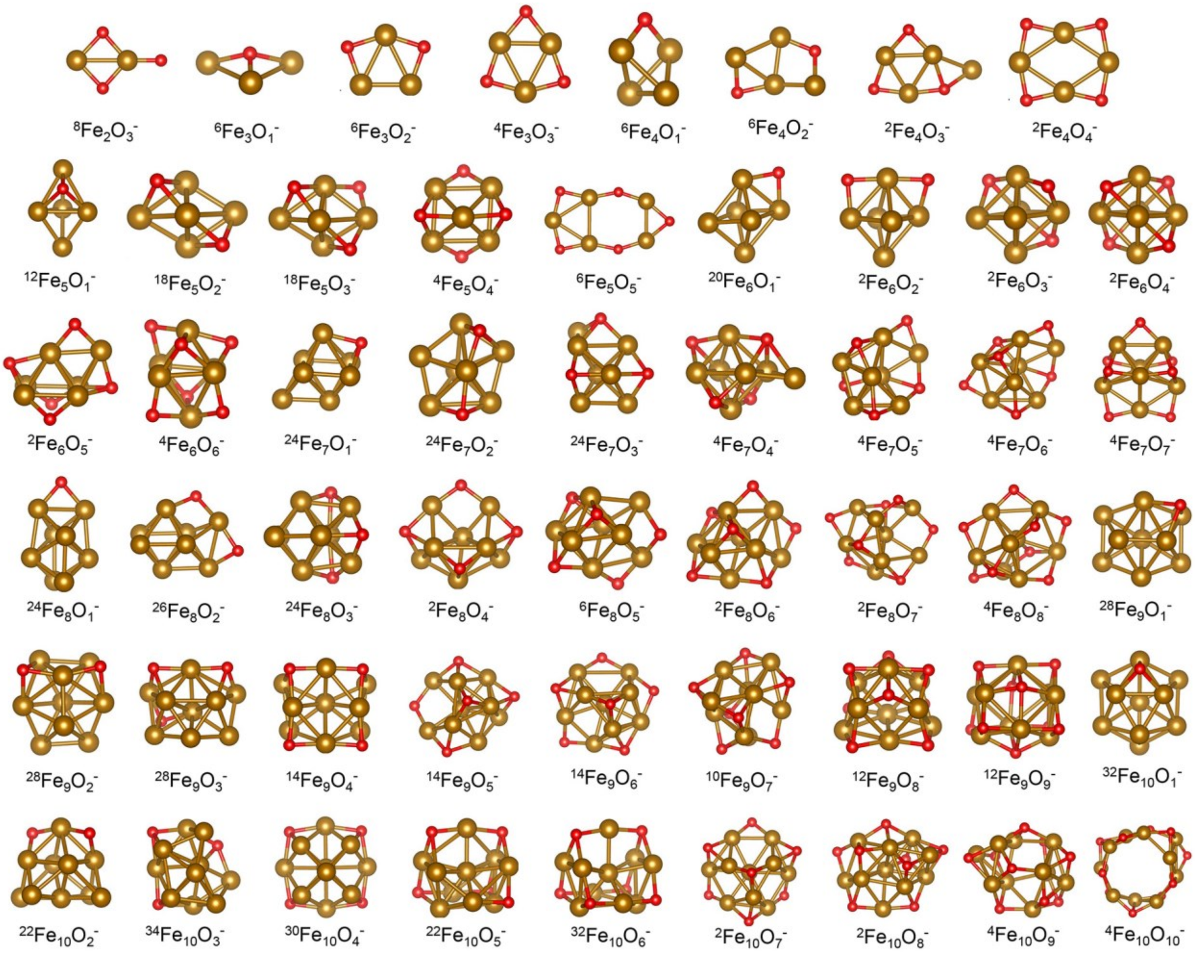

Supplementary Figure 6 | The lowest energy structures of typical $Fe_nO_m^-$ (n = 2-10) clusters, optimized at BPW91/6-311G(d) level of theory.

Supplementary Table 1 | The relative energies and spin multiplicities of $Fe_2O_3^-$ by DFT calculations at the BPW91/6-311G(d), BPW91/6-311+G(d), PBE/6-311+G(d), PBE/6-311G(d), B3LYP/6-311G(d), and B3PW91/6-311G(d) level of theory. The spin magnetic moment is also estimated for a comparison[11].

$Fe_2O_3^-$		BPW91/6-311G(d)	BPW91/6-311+G(d)	PBE/6-311G(d)	PBE/6-311+G(d)	B3LYP/6-311G(d)	B3PW91/6-311G(d)
Spin multiplicity	Spin magnetic moment (μ_B)	Relative energy (eV)	Relative energy (eV)	Relative energy (eV)	Relative energy (eV)	Relative energy (eV)	Relative energy (eV)
6	6.0	0.04	0.45	0.06	0.40	0.34	0.74
8	8.0	0	0	0	0	0	0
10	10.0	0.83	0.62	0.93	0.60	0.46	0.36

The electron-spin isomers

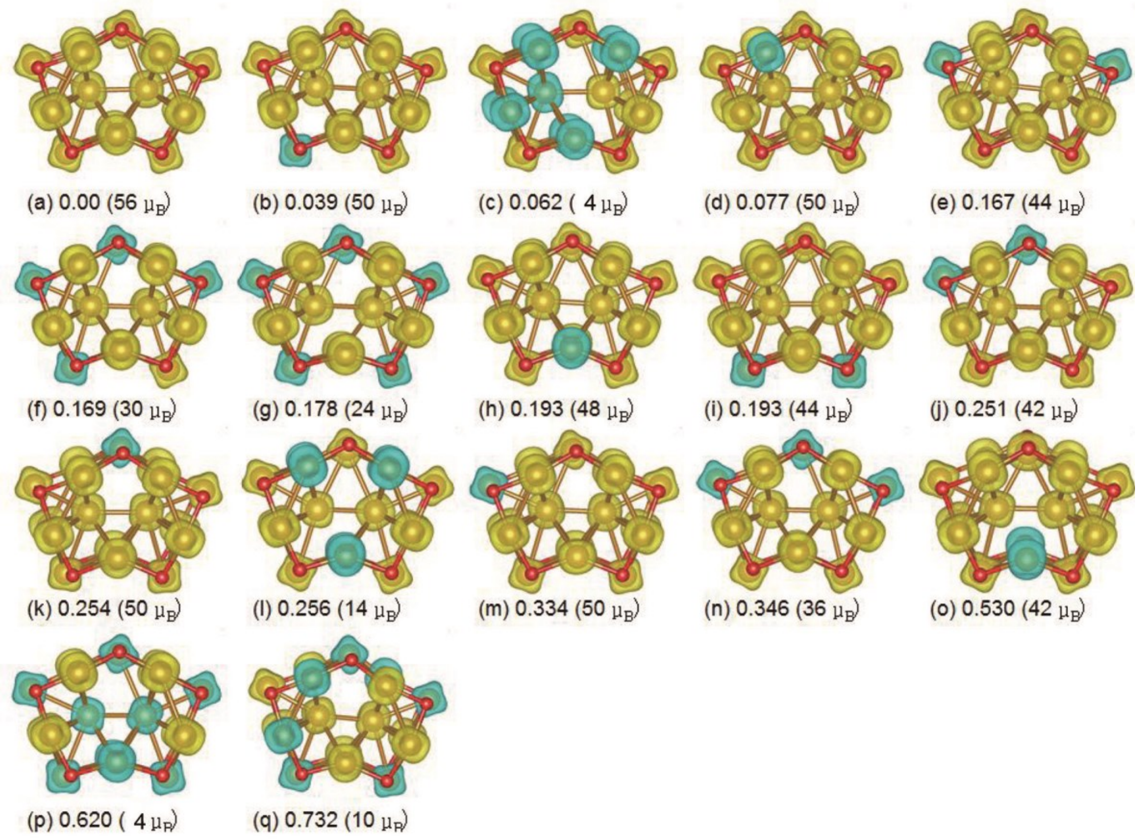

Supplementary Figure 7 | Electron-spin isomers of the $Fe_{17}O_{10}^{-}$ cluster with relative energy given in eV and magnetic moment in μ_B.

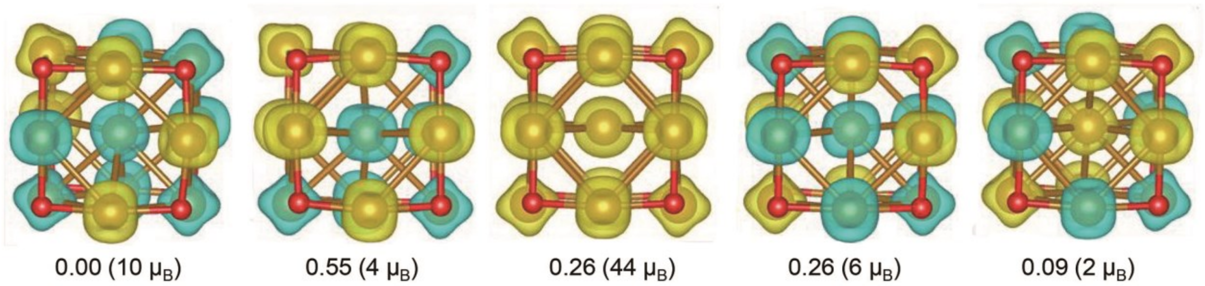

Supplementary Figure 8 | Electron-spin isomers of the $Fe_{13}O_{8}^{-}$ cluster with relative energy given in eV and magnetic moment in μ_B.

Geometry and AIMD simulations

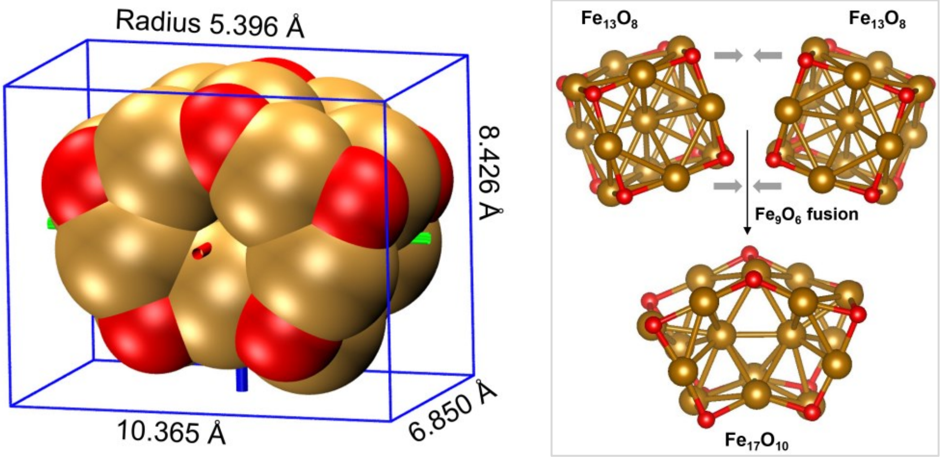

Supplementary Figure 9 | Geometry (Left) The van der Waals (VDW) cluster radius (given in Å) of the global minima of $Fe_{17}O_{10}^{-}$ cluster. **(Right)** $Fe_{17}O_{10}$ accordion-like structure through the Fe_9O_6 fusion from two $Fe_{13}O_8$ clusters.

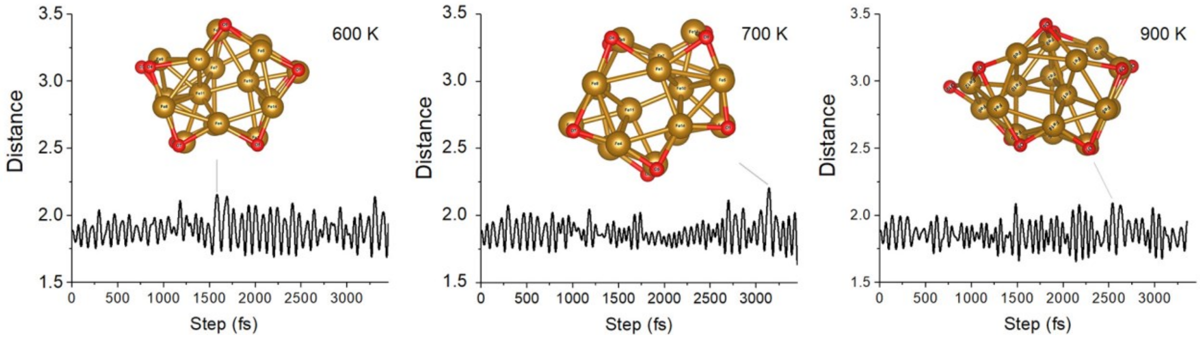

Supplementary Figure 10 | Molecular dynamics simulation. The AIMD simulations of $Fe_{17}O_{10}^{-}$ at 700 and 900 K for 3500 fs, with the Fe1-O4 distance indicated in Å. The time step was set to 1 fs.

ELF and NICS analyses

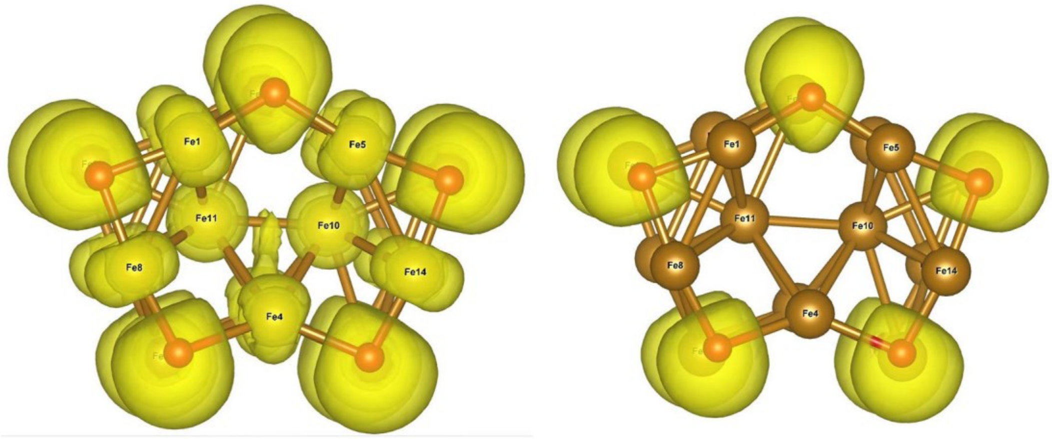

Supplementary Figure 11 | Calculated three-dimension electron localization function (3D-ELF) with an iso-surface value at 0.12 au. and 0.16 au., respectively, at the BPW91/6-311g(d) level of theory.

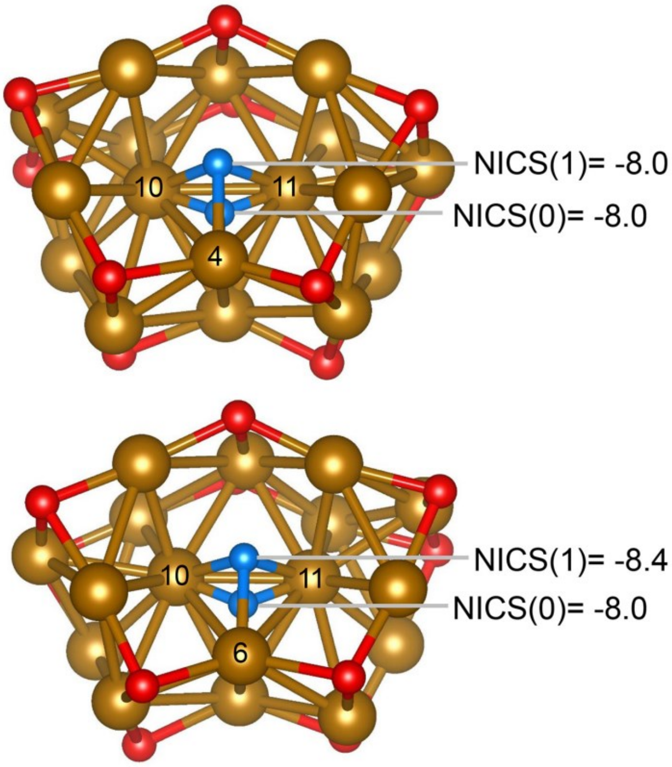

Supplementary Figure 12 | DFT-calculated NICS(0) and NICS(1) on the center and 1.0 Å above the Fe4-Fe10-Fe11, and Fe6-Fe10-Fe11 plane surface of the $Fe_{17}O_{10}^{-}$ cluster, respectively.

Bond lengths and NPA charge

Supplementary Table 2 | The Fe-O bond lengths in the $Fe_2O_2^-$, $Fe_4O_4^-$, $Fe_{13}O_8^-$, and $Fe_{17}O_{10}^-$ clusters, given in Å.

$Fe_2O_2^-$	Bond length (Å)	$Fe_4O_4^-$	Bond length (Å)	$Fe_{13}O_8^-$	Bond length (Å)	$Fe_{17}O_{10}^-$	Bond length (Å)
Fe1-O4	1.78177	Fe1-O6	1.76518	Fe1-O16	1.88287	Fe5-O18	1.86330
Fe2-O4	1.83500	Fe4-O6	1.76072	Fe4-O16	1.90736	Fe14-O18	1.87016
Fe1-O3	1.78177	Fe3-O5	1.76518	Fe6-O16	1.91000	Fe17-O18	1.88264
Fe2-O3	1.83497	Fe4-O5	1.76072	Fe4-O17	1.89377	Fe5-O26	1.83845
Average	1.80838	Fe3-O8	1.76529	Fe2-O17	1.93699	Fe1-O26	1.83354
		Fe2-O8	1.76055	Fe13-O17	1.90280	Fe13-O26	1.92562
		Fe1-O7	1.76529	Fe2-O18	1.92864	Fe1-O21	1.87733
		Fe2-O7	1.76055	Fe3-O18	1.90279	Fe9-O21	1.88010
		Average	1.76294	Fe12-O18	1.88647	Fe8-O21	1.85534
				Fe3-O20	1.90555	Fe8-O24	1.82030
				Fe1-O20	1.90876	Fe4-O24	1.98102
				Fe11-O20	1.89096	Fe12-O24	1.85386
				Fe7-O14	1.91114	Fe4-O22	1.93113
				Fe8-O14	1.89232	Fe14-O22	1.83249
				Fe13-O14	1.90811	Fe16-O22	1.90092
				Fe7-O15	1.89235	Fe16-O19	1.90029
				Fe6-O15	1.91891	Fe15-O19	1.83268
				Fe9-O15	1.91789	Fe6-O19	1.93121
				Fe8-O19	1.92261	Fe6-O27	1.98052
				Fe10-O19	1.90202	Fe2-O27	1.82043
				Fe12-O19	1.91801	Fe12-O27	1.85407
				Fe10-O21	1.90351	Fe2-O20	1.85532
				Fe11-O21	1.91923	Fe7-O20	1.87750
				Fe9-O21	1.92816	Fe9-O20	1.88015
				Average	1.90797	Fe7-O25	1.83326
						Fe13-O25	1.92577
						Fe3-O25	1.83846
						Fe3-O23	1.86267
						Fe15-O23	1.87040
						Fe17-O23	1.88302
						Average	1.87640

Supplementary Table 3 | NPA charge distribution of $Fe_2O_2^-$, $Fe_4O_4^-$, $Fe_{13}O_8^-$, and $Fe_{17}O_{10}^-$.

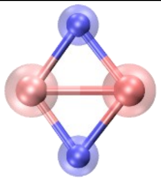 $Fe_2O_2^-$		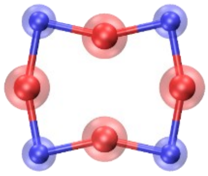 $Fe_4O_4^-$		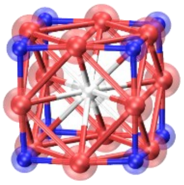 $Fe_{13}O_8^-$		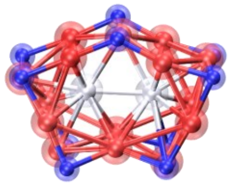 $Fe_{17}O_{10}^-$	
	Charge		Charge		Charge		Charge
Fe1	0.35680	Fe1	0.76247	Fe1	0.70987	Fe1	0.74340
Fe2	0.47852	Fe2	0.69908	Fe2	0.73506	Fe2	0.77286
O3	-0.91766	Fe3	0.76247	Fe3	0.70585	Fe3	0.80108
O4	-0.91766	Fe4	0.78083	Fe4	0.69839	Fe4	0.72191
Total	-1.00000	O5	-1.00713	Fe5	-0.24094	Fe5	0.80092
		O6	-1.00713	Fe6	0.69327	Fe6	0.72208
		O7	-0.99529	Fe7	0.71443	Fe7	0.74311
		O8	-0.99529	Fe8	0.72052	Fe8	0.77251
		Total	-1.00000	Fe9	0.71528	Fe9	0.70631
				Fe10	0.72983	Fe10	-0.33032
				Fe11	0.68579	Fe11	-0.32233
				Fe12	0.69457	Fe12	0.67803
				Fe13	0.70418	Fe13	0.59366
				O14	-1.16757	Fe14	0.68274
				O15	-1.15053	Fe15	0.68195
				O16	-1.15352	Fe16	0.65214
				O17	-1.15371	Fe17	0.68131
				O18	-1.16191	O18	-1.10062
				O19	-1.15689	O19	-1.09963
				O20	-1.15906	O20	-1.10632
				O21	-1.16291	O21	-1.10617
				Total	-1.00000	O22	-1.09976
						O23	-1.10060
						O24	-1.10923
						O25	-1.13479
						O26	-1.13488
						O27	-1.10935
						Total	-1.00000

O_2-binding analysis

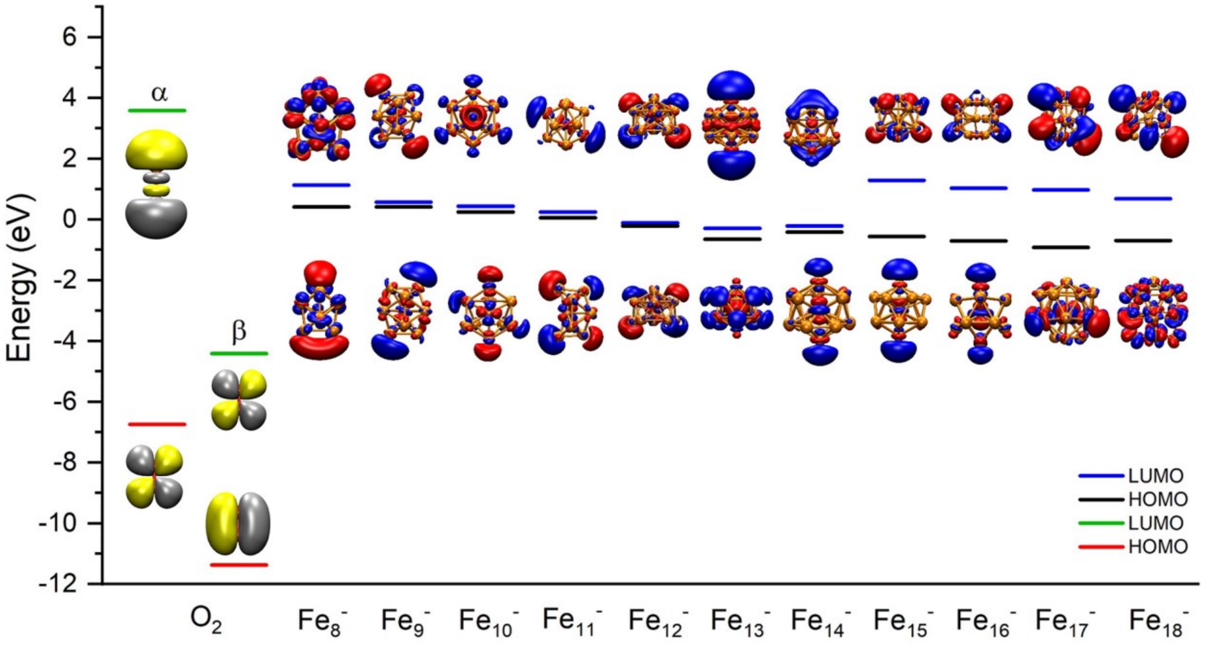

Supplementary Figure 13 | The calculated frontier orbitals of the O_2 and the Fe_n^- (n = 8−18) clusters at BPW91/6-311g(d) level of theory.

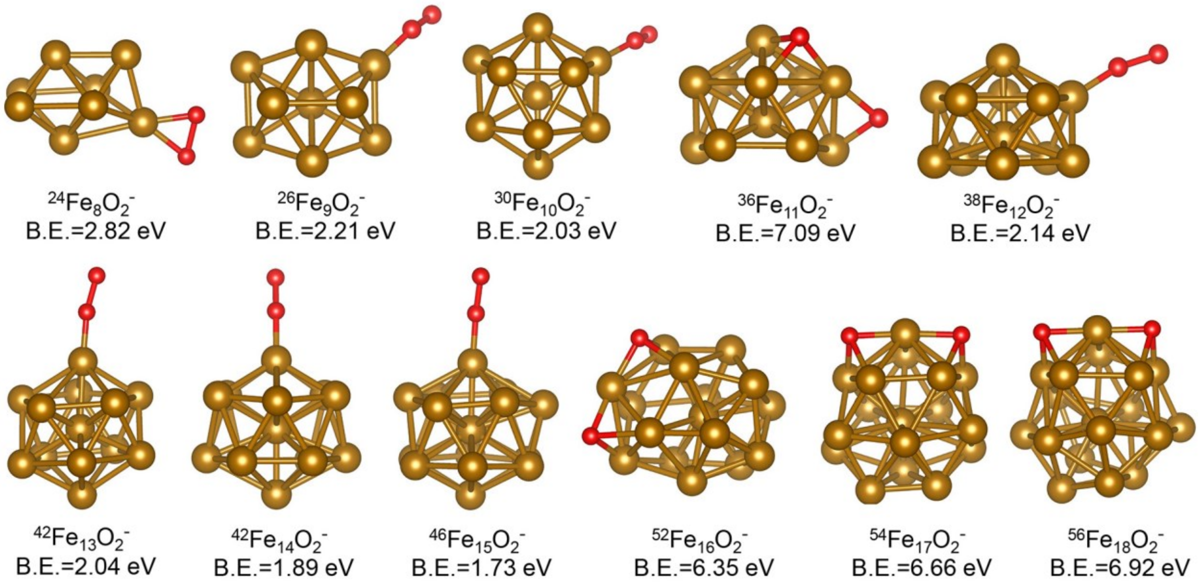

Supplementary Figure 14 | The optimized lowest energy structures of the $Fe_n{\cdot}O_2^-$ (n = 8−18) clusters at the BPW91/6-311g(d) level of theory. The presuperscripts refer to spin multiplicity. The O_2-binding energy (B.E.) are given in eV.

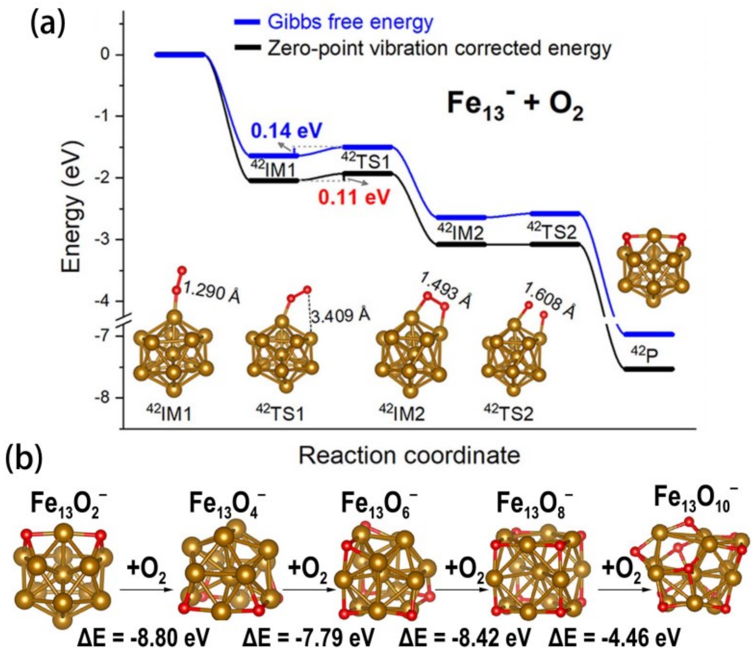

Supplementary Figure 15 | The reaction energy diagram for Fe_{13}^- with O_2. a, A comparison of the thermal corrected Gibbs free energies and the zero-point vibration corrected thermodynamic energies, calculated at the BPW91/6-311g(d) level of theory. **b**, The thermodynamic energy changes from $Fe_{13}O_2^-$ to $Fe_{13}O_{10}^-$ successively.

Supplementary References

1. Blochl, P. E. Projector augmented-wave method. *Phys. Rev. B*, **50**, 17953-17979 (1994).
2. Kresse, G. & Furthmüller, J. Efficiency of ab-initio total energy calculations for metals and semiconductors using a plane-wave basis set. *Comp. Mater. Sci.*, **6**, 15-50 (1996).
3. Kresse, G. & Furthmüller, J. Efficient iterative schemes for ab initio total-energy calculations using a plane-wave basis set. *Phys. Rev. B*, **54**, 11169-11186 (1996).
4. Kresse, G. & Hafner, J. Ab initio molecular-dynamics simulation of the liquid-metal-amorphous-semiconductor transition in germanium. *Phys. Rev. B*, **49**, 14251-14269 (1994).
5. Perdew, J. P., Burke, K. & Ernzerhof, M. Generalized gradient approximation made simple. *Phys. Rev. Lett.*, **77**, 3865-3868 (1996).
6. Palotás, K., Andriotis, A. N. & Lappas, A. Structural, electronic, and magnetic properties of nanometer-sized iron-oxide atomic clusters: Comparison between GGA and GGA+U approaches. *Phys. Rev. B*, **81**, 075403 (2010).
7. Yu, Y. L., Lim, K. H., Wang, J. Y. & Wang, X. CO Adsorption Behavior on Decorated Pt@Au Nanoelectrocatalysts: A Combined Experimental and DFT Theoretical Calculation Study. *J. Phys. Chem. C*, **116**, 3851-3856 (2012).
8. S. L. Dudarev, G. A. Botton, S. Y. Savrasov, Humphreys, C. J. & Sutton, A. P. Electron-energy-loss spectra and the structural stability of nickel oxide: an LSDA+U study. *Phys. Rev. B*, **57**, 1505-1509 (1998).
9. Zhang, H., Wu, H., Jia, Y., Geng, L., Luo, Z., Fu, H. & Yao, J. An integrated instrument of DUV-IR photoionization mass spectrometry and spectroscopy for neutral clusters. *Rev. Sci. Instrum.*, **90**, 073101 (2019).
10. Gutsev, G. L., Weatherford, C. A., Jena, P., Johnson, E. & Ramachandran, B. R. Structure and properties of Fe_n, Fe_n^-, and Fe_n^+ clusters, n = 7-20. *J. Phys. Chem. A*, **116**, 10218-10228 (2012).
11. Housecroft, C. E. & Sharpe, A. G. Inorganic Chemistry. 700-702 (pearson, 2012).
